# Do foundation doctors meet the mental health competencies in the foundation programme curriculum?

**DOI:** 10.1192/bjb.2019.81

**Published:** 2020-02

**Authors:** Jack Haywood

**Affiliations:** Academic Foundation Doctor, East and North Hertfordshire NHS Trust. email: jack.haywood@doctors.org.uk

In recent years, the UK Foundation Programme has been adapted in order to meet the changing demands of patients, as well as those of foundation year doctors. A broadening of the Foundation Programme in 2014 stipulated that 100% of foundation year doctors should undertake a placement including community care from August 2017.^1^ Further to this, in 2015, Health Education England set a target that 45% of foundation year trainees should complete a psychiatry placement to gain exposure to mental healthcare.^2^ However, it is important to recognise that mental health problems do not present just in the psychiatry setting. General practice (GP) and accident and emergency (A&E) are arguably ‘gateways’ to accessing mental healthcare in the National Health Service.

The Foundation Programme Curriculum 2016 outlines the expectations for what foundation doctors should learn. With the changes to community placements outlined above, I conducted a study to assess whether doing a foundation year placement in psychiatry, A&E and/or GP affects trainees’ ability to meet the Foundation Programme mental health competencies. I used a cross-sectional questionnaire to ask foundation year 2 (FY2) and CT1/ST1 trainees whether they felt they had met 17 mental health-related competencies from the curriculum.^3^ This was sent electronically via Foundation Schools.

A total of 360 trainees took the survey. Of all the trainees, only 29.7% (n = 107) were aware that there are specific mental health competencies in the Foundation Programme curriculum that they should have met. Fourteen of the 17 competencies were self-assessed to have been met by trainees who completed a placement in psychiatry, compared with 15 out of 17 in A&E and 13 out of 17 in GP. By comparison, only nine competencies out of 17 were met by those who did not have a placement in any of the three specialties. Interestingly, in all groups, the competencies that should have been met by the end of FY1 were more successfully met than those for FY2.

These results were for trainees who may have taken one, two or all three specialties in their foundation year training. When considering each specialty uniquely, ten out of 17 competencies were met in psychiatry, whereas only six were met in A&E and nine in GP.

These results suggest that a combination of these three specialties may be more beneficial than one specialty alone, or none at all, in order for trainees to feel they have met the mental health competencies. There is also a case to be argued that trainees should be educated about the curriculum, as many were not aware they had the competencies to meet.

Further research should involve asking assessors to make the same competency assessments about the foundation year trainees, moving away from self-assessment to a work-based assessment.
